# Effects of aging on the quality of roasted sesame-like flavor *Daqu*

**DOI:** 10.1186/s12866-020-01745-3

**Published:** 2020-03-26

**Authors:** Guangsen Fan, Zhilei Fu, Chao Teng, Pengxiao Liu, Qiuhua Wu, Md Khondakar Raziur Rahman, Xiuting Li

**Affiliations:** 1 Beijing Advanced Innovation Center for Food Nutrition and Human Health, Beijing Technology and Business University (BTBU), Beijing, 100048 China; 2 School of Food and health, Beijing Technology and Business University (BTBU), Beijing, 100048 China; 3 Beijing Engineering and Technology Research Center of Food Additives, Beijing Technology & Business University (BTBU), No 11 Fucheng Street, Haidian District, Beijing, 100048 China

**Keywords:** *Baijiu*, Esterifying activity, Eukaryotic microbes, High-throughput sequencing, Prokaryotic microbes, Volatile compounds

## Abstract

**Background:**

*Daqu*, the saccharification, fermentation, and aroma-producing agents for *Baijiu* brewing, is prepared using a complex process. Aging is important for improving the quality of *Daqu*, but its impact has rarely been studied. This study investigated changes in the physicochemical properties, flavor compounds, and microbial communities during aging of *Daqu* with a roasted sesame-like flavor.

**Results:**

The physicochemical properties changed continuously during aging to provide a high esterifying activity. Aging removed unpleasant flavor compounds and helped to stabilize the flavor compounds in mature *Daqu*. A high-throughput sequencing approach was used to analyze the changing composition of the microbial communities during aging. Aging helped to modify the microbial population to produce better *Baijiu* by eliminating low-abundance microbial communities and optimizing the proportion of predominant microbial communities. Nine genera of prokaryotic microbes formed the core microbiota in *Daqu* after aging. Regarding eukaryotic microbes, Zygomycota, the predominant community, increased in the first 2 months, then decreased in the third month of aging, while Ascomycota, the subdominant community, showed the opposite behavior. *Absidia*, Trichocomaceae_norank and *Rhizopus* were the predominant genera in the mature *Daqu*.

**Conclusions:**

Significant correlations between microbiota and physicochemical properties or flavor compounds were observed, indicating that optimizing microbial communities is essential for aging *Daqu*. This study provides detailed information on aging during *Daqu* preparation.

## Background

*Baijiu*, one of the most famous traditional distilled spirits in the world, is produced by the spontaneous solid-state fermentation of a sorghum, wheat, and/or rice mixed-culture, a process which mainly involves *Daqu* manufacture and grain fermentation [1, 2]. *Daqu*, the saccharifying and fermenting agent used for brewing *Baijiu*, is critical for obtaining the final flavor characteristics of *Baijiu* [3]. *Daqu* acts not only as a resource for the brewing process, but also provides important crude enzymes, complex microbial flora, and even aroma precursors that will determine the flavor characteristics of the final liquor product [4]. Therefore, the contribution of *Daqu* to *Baijiu* manufacture has been summarized by brewers in the expression “better *Daqu*, better *Baijiu*” [5].

**Fig. 1:**
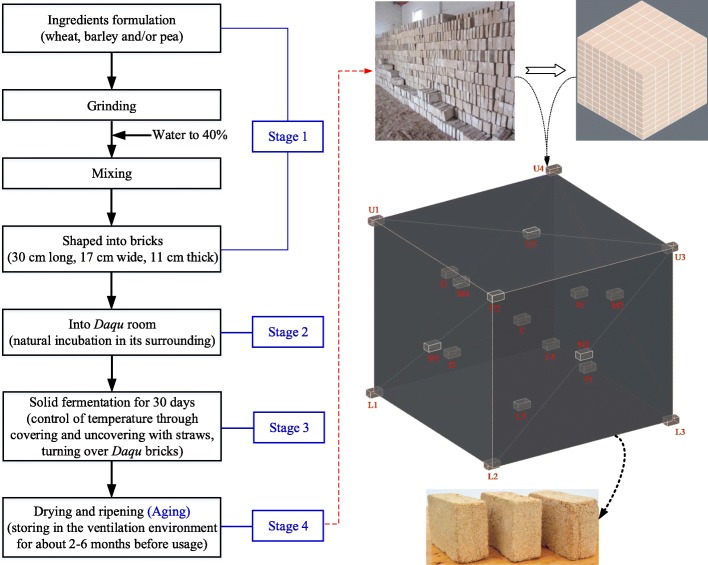
Process flow diagram for the production of *Daqu* and the diagrammatic drawing of sampling for *Daqu* samples

*Daqu* is known as the heart of *Baijiu* owing to its importance in the process. Similar to *Baijiu*, *Daqu* is made from wheat, barley, and/or peas and formulated through a complex solid-state fermentation in an open environment. The process includes preparation of the ingredients, grinding, mixing, shaping, incubation, spontaneous solid fermentation for 1 month, and a long aging period (drying and ripening) to allow the *Daqu* to reach maturity (Fig. 1) [6]. Each of these stages has a significant impact on *Daqu* quality. Owing to differences in these stages and production environments, different types of *Daqu* with different characteristics are produced, such as Jiang-, Nong-, Qing-, and roasted sesame-like flavor *Daqu*, characterized by flavor, or low-temperature, medium-temperature, and high-temperature *Daqu*, characterized by the maximum incubation temperature [7–9]. Each type of *Daqu* has a unique combination of microbiota dynamics, functional enzymes, and special flavor that are essential in *Baijiu* production as saccharification, fermentation, and aroma-producing agents [2, 9, 10]. For example, *Bacillus* species are predominant in Nong- and Jiang-like flavor *Daqu*, while lactic acid bacteria (LAB) are dominant in all types of *Daqu* [10]. Furthermore, *Thermomyces lanuginosus* is dominant in Jiang-like flavor *Daqu*, while *Saccharomycopsis fibuligera* and *Lichtheimia ramose* occur in Qing- and Nong-like flavor *Daqu* [11]. In other words, no single genus has been found in all types of *Daqu*. For example, *Aspergillus*, *Gilmaniella*, *Monascus*, *Penicillum*, *Mucor*, *Trichoderma*, *Rhizomucor*, *Absidia* and *Rhizopus* are common fungi in *Daqu*, but are not present in all types of *Daqu*, while each type of *Daqu* does not contain all the above fungi [9]. The differences in microbial communities between different types of *Daqu* are related to the environmental conditions and production processes [2, 7].

Aging, the last step in the process of *Daqu* preparation, seems to be simple procedure, in which fermented *Daqu* is placed somewhere open, cool, well-ventilated, and dry for a given period of time. In fact, from thousands of years of *Daqu* production experience, aging is known to plays a particularly important role in achieving high-quality *Daqu*. After fermentation, *Daqu* is usually stored for at least 2 months before being used to make *Baijiu* [12]. During this time, according to experienced production workers, *Daqu* is “purified” by “removing discordant” substances, and its “fragrance increases” [13]. Only *Daqu* that has been aged for a reasonable period can produce high-quality *Baijiu*. With recent developments in analytical technologies, such as high-throughput sequencing (HTS) and headspace solid-phase microextraction–gas chromatography–mass spectrometry (HS-SPME-GC-MS), researchers have discovered that the aging process when making *Daqu* is not simply affected by storage time, but also by a very complicated process in which the microbial flora constantly change with changes in the external environment, such as moisture and temperature. The microorganisms in *Daqu* after fermentation also remain active, causing changes in its enzyme and metabolite contents. Therefore, aging can encourage the enriched microbes to interact further, such that the mixed microbial communities achieve a balance that helps to stabilize the quality of the final *Baijiu* product [14]. Aging is critical for making *Daqu*, as it affects its quality, which, in turn, influences the quality of *Baijiu*. Accordingly, only well-aged *Daqu* can be used to make *Baijiu*, making the end point of *Daqu* storage an important control point in *Daqu* preparation.

Presently, decisions regarding whether the quality of *Daqu* is sufficient for use in *Baijiu* production depends mainly on sensory evaluation according to accumulated production experience and partly on its physicochemical properties, which can be subjective and uncertain. This leads to an unstable level of quality in the *Baijiu* produced. With the increase in *Baijiu* production and a reduction in the number of workers engaged in its production, *Daqu* manufacture has evolved from the original simple artisanal scale into a large-scale industrial process to keep up with demand [15]. During this transformation process, it is necessary to clearly understand the internal mechanisms during each stage of *Daqu* production. However, the effects of aging during *Daqu* manufacture have yet to be explained scientifically, which is a problem that needs to be solved to promote a successful transformation from the artisanal to large-scale production of *Daqu*, as well as its effect on *Baijiu* production.

The process of *Daqu* preparation involves collecting functional microbes suitable for *Baijiu* production, such that the composition of the microbial communities and their abundance can undergo complex changes during the entire production process. Changes in microbial activity also cause changes in the physicochemical properties (especially enzymatic activity) and flavor substances of *Daqu*. Therefore, fully understanding the changing role of the microbial community, physicochemical properties, and flavor substances might provide a more comprehensive scientific explanation of the necessity of each step during the *Daqu* production process. The process of aging is important for microbial growth and the flavor compounds produced in *Daqu*. However, few studies have analyzed the dynamics of the microbiota, flavor compounds, and physicochemical properties of *Daqu* at this stage, with no scientific methods available for determining whether *Daqu* was mature. Therefore, analyzing these dynamics can provide a better understanding of the contribution of aging to the quality of *Daqu*, especially in providing a scientific basis for judging when *Daqu* reaches maturity during storage.

Therefore, this study used roasted sesame-like flavor (RSF) *Daqu* as the specific research object to investigate changes in its microbial communities and the content of flavor substances during aging, using HTS and HP-SPME-GC-MS, respectively. The physicochemical properties will be investigated to determine the potential role of the microorganisms in the *Daqu*. To our knowledge, this is the first comprehensive study to report the effects of aging during RSF *Daqu* manufacture on its physicochemical properties, flavor substances, and microbial flora.

## Results and discussion

### Physicochemical properties

As shown in Table 1, all physicochemical parameters of *Daqu* changed during aging, except for fermenting activity. During aging, the moisture content of *Daqu* decreased as aging proceeded. This was caused by the *Daqu* being stored in a relatively open well-ventilated environment with dry, cool air (Fig. 1). The contents of protein and starch in the *Daqu* tended to increase during storage. Two possible explanations for this were that moisture in the *Daqu* decreased significantly during aging, which increased the contents in terms of concentration, or that enrichment of the microbial communities and changes in some microbes were partly responsible [14]. For example, in the subsequent analysis of microbial strains, the numbers of *Cyanobacteria* increased with storage time, which might lead to the increase in protein and starch contents, because this genus synthesized starch and protein through photosynthesis [16]. Owing to the microbial activity, especially in acid-producing microorganisms such as *Acetobacter* and *Lactobacillus*, the acidity and pH fluctuated during aging (Table 1), After 2–3 months, the acidity of the *Daqu* became more stable. Presently, acidity is an important criterion used to judge whether *Daqu* is mature, and is the only objective standard used during the current *Daqu* preparation process [17]. After aging for 3 months, the saccharifying and liquefying activities had reduced significantly from the initial values, while the esterifying activity had increased. This was probably caused by changes in the microbial flora during aging. Some microorganisms that produce lipase and other enzymes related to ester synthesis increased, while others mainly producing amylase and glucoamylase decreased. These differences in enzyme production might have led to the changes in temperature during the process of brewing high quality *Baijiu*, which conforms to the principle of “slow rise in the early stage, rapid rise in the middle, and slow fall in the later stage”, as summarized in the *Baijiu* brewing process [18]. Interestingly, the fermenting activity did not change significantly during aging, mainly due to the balance of interactions between the various microorganisms.

**Table 1:** Changes in physicochemical properties of RSF *Daqu* during aging

Sample	Moisture (%)	Protein (%)	Starch (%)	Acidity (mmol g^−1^)	pH	Saccharifying activity (U)	Liquefying activity (U)	Esterifying activity (U)	Fermenting activity (U)
A0	12.91 ± 0.03^c^	15.11 ± 0.19^a^	50.71 ± 0.47^a^	0.39 ± 0.02^a^	6.75 ± 0.03^c^	849.00 ± 4.24^d^	0.48 ± 0.01^c^	407.54 ± 2.01^b^	0.30 ± 0.07^a^
A1	8.07 ± 0.07^b^	16.40 ± 0.15^b^	56.23 ± 0.43^b^	0.68 ± 0.04^c^	6.51 ± 0.00^a^	783.00 ± 4.24^c^	0.44 ± 0.01^b^	293.23 ± 13.01^a^	0.27 ± 0.02^a^
A2	8.04 ± 0.01^b^	16.70 ± 0.06^c^	58.23 ± 0.11^c^	0.50 ± 0.00^b^	6.86 ± 0.00^d^	706.50 ± 2.12^b^	0.34 ± 0.00^a^	526.82 ± 6.02^c^	0.23 ± 0.02^a^
A3	6.44 ± 0.01^a^	17.06 ± 0.02^d^	60.46 ± 0.47^d^	0.52 ± 0.11^b^	6.60 ± 0.00^b^	672.00 ± 8.49^a^	0.32 ± 0.01^a^	589.30 ± 2.01^d^	0.23 ± 0.09^a^

Note: Same letters in the column do not differ significantly at 5% probability by Tukey test. A0, at the beginning of aging; A1, after 1 month of aging; A2, after 2 months of aging; A3, after 3 months of aging

### Flavor compounds in *Daqu*

In the present study, a total of 34 volatile compounds, namely, six alcohols, seven esters, six aldehydes, three benzodiazepines, one nitrogen-containing compound, five ketones, one phenol, four alkanes, and one alkene, were identified in *Daqu* using HS-SPME-GC-MS (Additional file 1: Table S1). In general, the relative abundance of the volatile compounds changed during aging because of the changes in microbial flora and their activity [19]. Overall, the alcohols, aldehydes, nitrogen-containing compounds, and alkenes increased slightly in concentration, while the concentrations of benzodiazepines, ketones, phenols, and alkanes decreased. However, the concentrations of the esters remained relatively unchanged. Similar to previous results, the concentrations of various flavor compounds had changed, but they all tended to be stable during aging [14, 20]. This might provide a scientific basis for determining the appropriate aging treatment of *Daqu* through sensory evaluation [17]. Regarding a single important group of flavor substances, the content of phenethyl alcohol with its rose scent, 1-octen-3-ol with its roasted mushroom aroma, and some biologically active compounds, such as ledol, tetramethylpyrazine, and caryophyllene, had increased during aging. Therefore, the aging stage of making *Daqu* has played an essential role in removing discordant flavors from *Daqu* and forming the unique full-bodied “*Daqu*-flavor” [20].

### Overall structure and diversity of prokaryotic communities

**Fig. 2:**
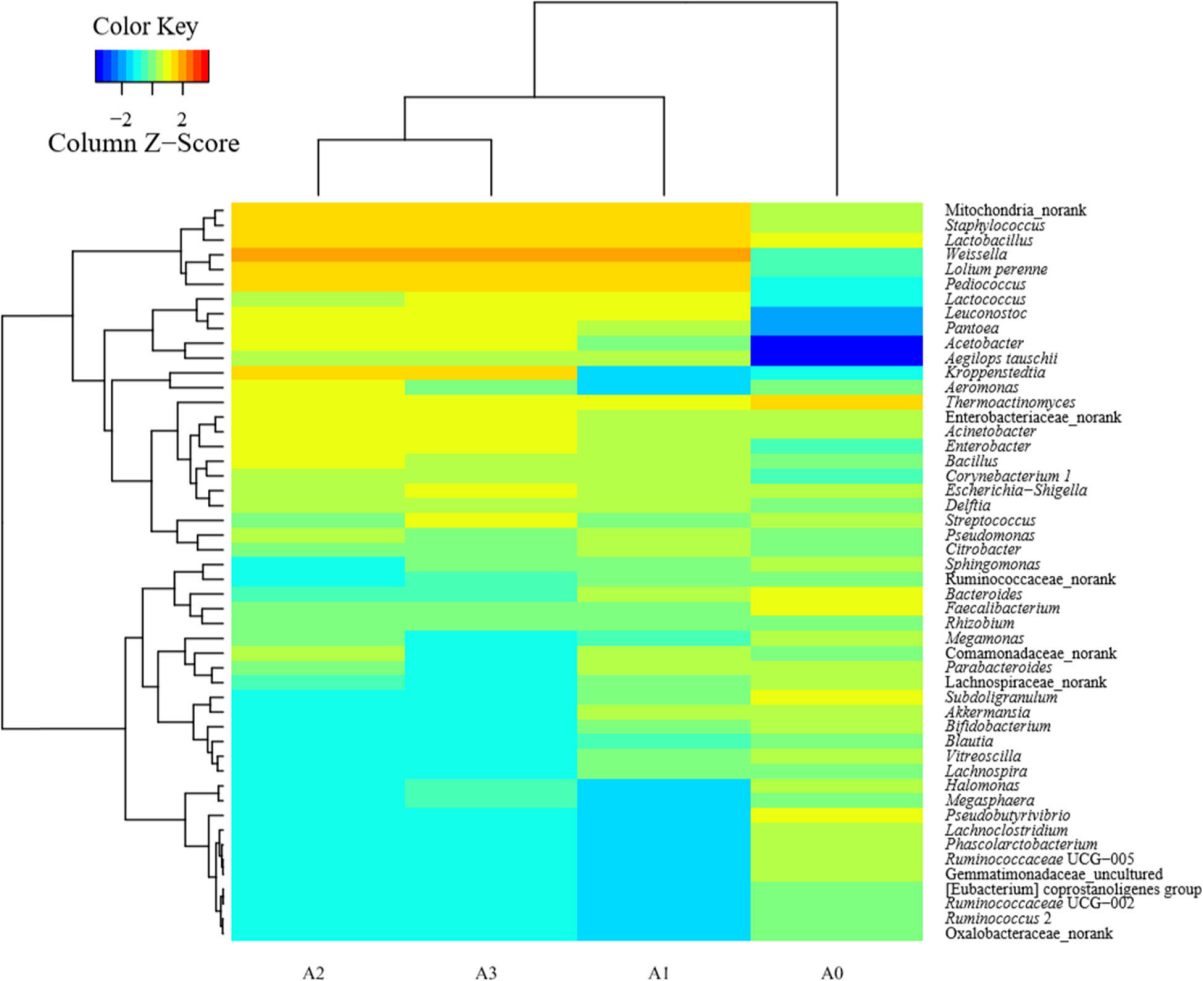
Taxonomic classification of sequences from prokaryotic communities of four samples (A0, at the beginning of aging; A1, after 1 month of aging; A2, after 2 months of aging; A3, after 3 months of aging) at genus level. The relative abundance was calculated by dividing the number of classified tags by the total tags number of each sample. The top 50 genera detected in RSF *Daqu* are shown. The relative abundance of each genus was indicated by color intensity in a heat map

For prokaryotic microbes, 587,885 valid sequencing reads with an average read length of 452 bp were obtained from all *Daqu* samples (Table 2). Rarefaction analysis indicated that all prokaryotic communities were well-represented at the sequencing depth, as the rarefaction curves approached a clear-cut asymptotic plateau. A total of 40 phyla and 733 genera were obtained based on 97% similarity as a cutoff in the 16S rRNA sequences. The richness and evenness of prokaryotic communities in *Daqu* were evaluated using α-diversity metrics, including the Chao 1, ACE, Shannon and Simpson indices. The richness estimators (Chao 1 and ACE) were consistent with the Shannon indices (Table 2). All indices showed that the microbial richness of *Daqu* decreased during the first month of aging, increased after 2 months, and then stabilized until the end of aging, indicating that aging-time-related changes had occurred at the OTU level. Based on the relative abundances of OTUs and taxonomic ranks from genus to OTU level, the prokaryotic communities in the four *Daqu* samples formed three clusters, namely, group I, sample A0; group II, sample A1; and group III, samples A2 and A3 (Fig. 2). Similarly, UniFrac clustered analysis and PCoA analysis showed that the prokaryotic communities could be clustered into three groups (Additional file 2: Figs. S1 and S2). These results showed that the differences between *Daqu* samples decreased during aging.

**Table 2:** Summary of sequencing results and the alpha diversity statistical analysis of RSF *Daqu* samples

Type	Sample ID	Reads	0.97					
			OTU^a^	ACE	Chao1	Good’s coverage	Shannon	Simpson
Prokaryote	A0	58,641	3230	3277	3244	0.9972178	9.8000	0.9923
	A1	136,340	914	1077	1004	0.9956349	4.6513	0.9066
	A2	108,260	1304	1652	1510	0.99205719	5.2338	0.9280
	A3	284,644	1199	1572	1446	0.9922820	5.0543	0.9153
Eukaryon	A0	105,966	100	124	113	0.9995384	2.3974	0.7061
	A1	188,004	134	168	163	0.9992980	2.1960	0.6678
	A2	151,282	103	139	136	0.9993785	1.6651	0.4265
	A3	505,639	142	155	149	0.9995790	2.1225	0.6049

Note: A0, at the beginning of aging; A1, after 1 month of aging; A2, after 2 months of aging; A3, after 3 months of aging. ^a^ Operational taxonomic units (OTUs) were defined with 97% similarity level

**Fig. 3:**
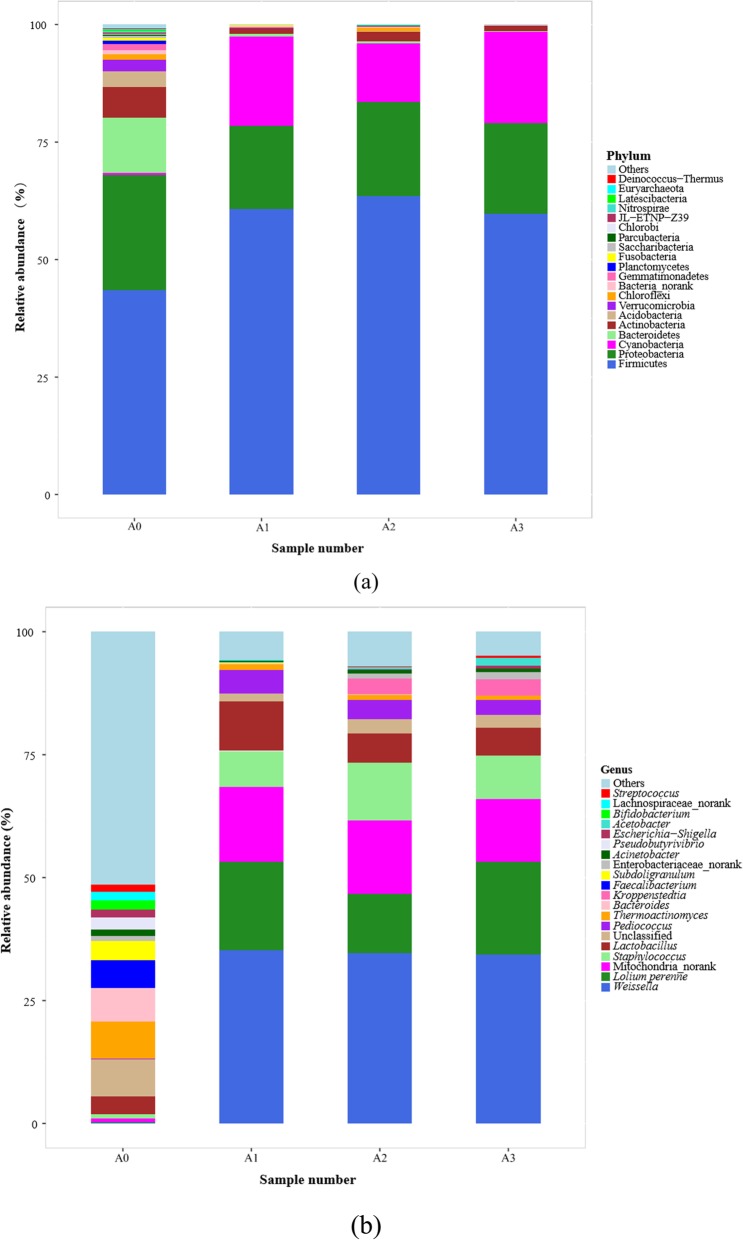
Analysis of prokaryotic communities composition in four samples (A0, at the beginning of aging; A1, after 1 month of aging; A2, after 2 months of aging; A3, after 3 months of aging) using high-throughput sequencing. Results of taxonomy at the phylum level and genus level are shown in (**a**) and (**b**), respectively. The relative abundance defines sequence percentages in samples as depicted by the colors in the bar chart. The top 20 phyla and genera detected in RSF *Daqu* are shown in (**a**) and (**b**), respectively. The rest of phyla and genera detected in all samples were classified as others

The taxonomic distribution at the phylum level is shown in Fig. 3a. More than 95% of the total abundances in each *Daqu* sample was precisely assigned to nine known bacterial phyla, namely, Firmicutes, Proteobacteria, Cyanobacteria, Bacteroidetes, Actinobacteria, Acidobacteria, Verrucomicrobia, Chloroflexi, and Gemmatimonadetes (Fig. 3a). Overall, during the *Daqu* aging process, the bacterial structure became optimized and the bacterial flora gradually stabilized. Specifically, Firmicutes, the predominant phylum accounting for 43.44% of the total prokaryotic reads before aging, increased to 60.73% after 1 month, then became stable at around 60% as aging progressed. Meanwhile, the proportion of reads for the subdominant phylum, Proteobacteria, fell slightly from 24.53 to 19.38%, before stabilizing. Bacteroidetes, the third most dominant phylum, accounted for 11.71% of total prokaryotic sequences at the beginning of aging, which decreased sharply as aging proceeded, accounting for only 0.01% after 3 months of aging. Other phyla, such as Actinobacteria, Acidobacteria, Verrucomicrobia, Chloroflexi, Gemmatimonadetes, Planctomycetes, Fusobacteria, Saccharibacteria, Parcubacteria, Nitrospirae, Latescibacteria, and Euryarchaeota, also decreased clearly. However, the increase in Cyanobacteria from 0.50% at the beginning of aging to 19.45% after 3 months of aging was more obvious, becoming the third most dominant phylum by the end of aging. This might explain why the starch content and protein content increased during aging. During aging, many low-abundance phyla were gradually eliminated, which could further increase the abundance of the dominant bacteria (Fig. 3). In short, aging is a process for further rebalancing the microorganisms in *Daqu*.

In general, the structure of bacteria in *Daqu* at the genus level at the beginning of aging was quite different to that after aging, but tended to be consistent with different aging times (Fig. 3b). At the beginning of aging, Unclassified (7.60%), *Thermoactinomyces* (7.57%), *Bacteroides* (6.84%), and *Faecalibacterium* (5.66%) were regarded as the predominant genera, with a relative abundance greater than 5%. *Subdoligranulum* (3.93%), *Lactobacillus* (3.61%), *Pseudobutyrivibrio* (2.43%), *Bifidobacterium* (1.89%), Lachnospiraceae_norank (1.81%), *Escherichia*-*Shigella* (1.60%), *Akkermansia* (1.37%), *Streptococcus* (1.36%), *Megamonas* (1.31%), *Sphingomonas* (1.29%), *Acinetobacter* (1.27%), and *Lachnoclostridium* (1.13%) were the subdominant genera (1% < subdominant < 5%) (Fig. 3b). As aging proceeded, the proportion of all above genera, except *Lactobacillus*, tended to decrease. After aging for a month, *Weissella*, Mitochondria_norank, *Lactobacillus*, and *Staphylococcus* increased to become the predominant genera, while *Pediococcus* increased, and Unclassified and *Thermoactinomyces* decreased, to become the subdominant genera. Compared with *Daqu* aged for 1 month, *Kroppenstedtia*, *Acetobacter*, and Enterobacteriaceae_norank increased to become the subdominant genera, while *Thermoactinomyces* decreased to become a subdominant genus at the end of aging. The proportions of the other genera decreased with aging time. Therefore, during aging, the microbial flora of *Daqu* were decontaminated by removing unwanted genera such as *Bdellovibrio*, and the proportion of functional microbes, which have an important role to play in the quality of *Baijiu*, was optimized. This might explain why the flavor compounds in *Daqu* were purified during aging.

Only properly aged *Daqu* can be used in *Baijiu* brewing, meaning that the composition of microorganisms in aged *Daqu* plays an important role in *Baijiu* manufacture. The core microbiota, comprising the dominant microbial genera, are generally considered to play the most important role in the process of *Baijiu* manufacture. In the present study, we identified nine genera of prokaryotic microbes, including *Weissella*, Mitochondria_norank, *Staphylococcus*, *Lactobacillus*, *Kroppenstedtia*, *Pediococcus*, Unclassified, *Acetobacter*, and Enterobacteriaceae_norank as the core microbiota in mature *Daqu*. Among these, three were LAB genera (*Weissella*, *Lactobacillus* and *Pediococcus*), meaning that LAB were one of the dominant bacterial genera in *Daqu*, consistent with previous studies [21]. LABs play a significant role in improving *Baijiu* quality, not only transform glucose or starch into lactic acid and keeping *Daqu* acidic, but also greatly contributing to providing substrates for the esterification of yeasts forming ester compounds [21, 22]. This may be important for explaining the increase in the esterifying activity of *Daqu* during aging. A previous study has shown that the contents of important esters in *Baijiu* are positively correlated with the numbers of LAB, indicating that a lag in fermentation and a lower ester content during *Baijiu* production could be caused by a lack of LAB [1]. Previous studies have shown that *Staphylococcus* is dominated in *Daqu*, with the potential to form amino acids, biogenic amines, aldehydes, free fatty acids, and esters [3, 23]. The number of *Staphylococcus* bacteria was correlated with the biosynthesis of the main organic acids in *Baijiu* [24]. Therefore, *Staphylococcus* has an important regulatory effect on the synthesis of four main esters (ethyl acetate, ethyl lactate, ethyl butyrate, and ethyl caproate), which greatly affects the quality of *Baijiu*. Few studies have focused on *Kroppenstedtia*, belonging to the family *Thermoactinomycetaceae*, which was isolated recently. *Kroppenstedtia* has been identified in several types of *Daqu*, namely, Baiyunbian high-temperature *Daqu*, light-flavor *Daqu*, and RSF *Daqu*, using high throughput sequencing [24–27]. Although its role in *Baijiu* manufacture is not clear, it is abundant at the beginning of fermentation but then decreased immediately, giving way to *Lactobacillus* [25]. *Acetobacter*, the prevalent bacteria during *Baijiu* production, greatly influences the flavor and taste of the final product, because it not only produces acetic acid, but also affords acetoin, a biosynthetic in *Baijiu* liquor [28]. *Acetobacter* can also select most of the acid-tolerant microbes owing to the high concentrations of acetic acid it produces [3]. In other words, *Acetobacter* can affect the microbial flora in the fermentation process of *Baijiu*, which will eventually affect the quality of the final product.

### Overall structure and diversity of eukaryotic communities

After removing the low quality chimera reads and trimming of the PCR primers, a total of 950,891 high-quality reads, with an average read length of 442 bp, corresponding to 18S rDNA gene sequences, was obtained (Table 2). The rarefaction curves reached the saturation plateau, indicating that the eukaryotic communities were well captured in the current analysis. In contrast to the prokaryotic community, the diversity of the eukaryotic microbes was smaller. Unlike the prokaryotic community, the eukaryotic Chao 1 estimator, ACE, and observed OTUs first increased, then fluctuated during aging, indicating that the succession of the eukaryotic communities was different from that of the prokaryotic communities during the whole aging process. The microbial richness (indicated by Chao 1 and ACE) of the eukaryotic community was highest in samples A1 and A3, followed by A2, with the lowest richness in sample A0. The microbial diversity (Shannon) of A0 was significantly higher than that of A2, while those of A1 and A3 had similar intermediate values (Table 2). PCoA analysis and heatmap results showed that sample A2 formed a cluster, and samples A0, A1 and A3 another large cluster with A1 and A3 being clustered together (Additional file 2: Figs. S3 and S4).

**Fig. 4:**
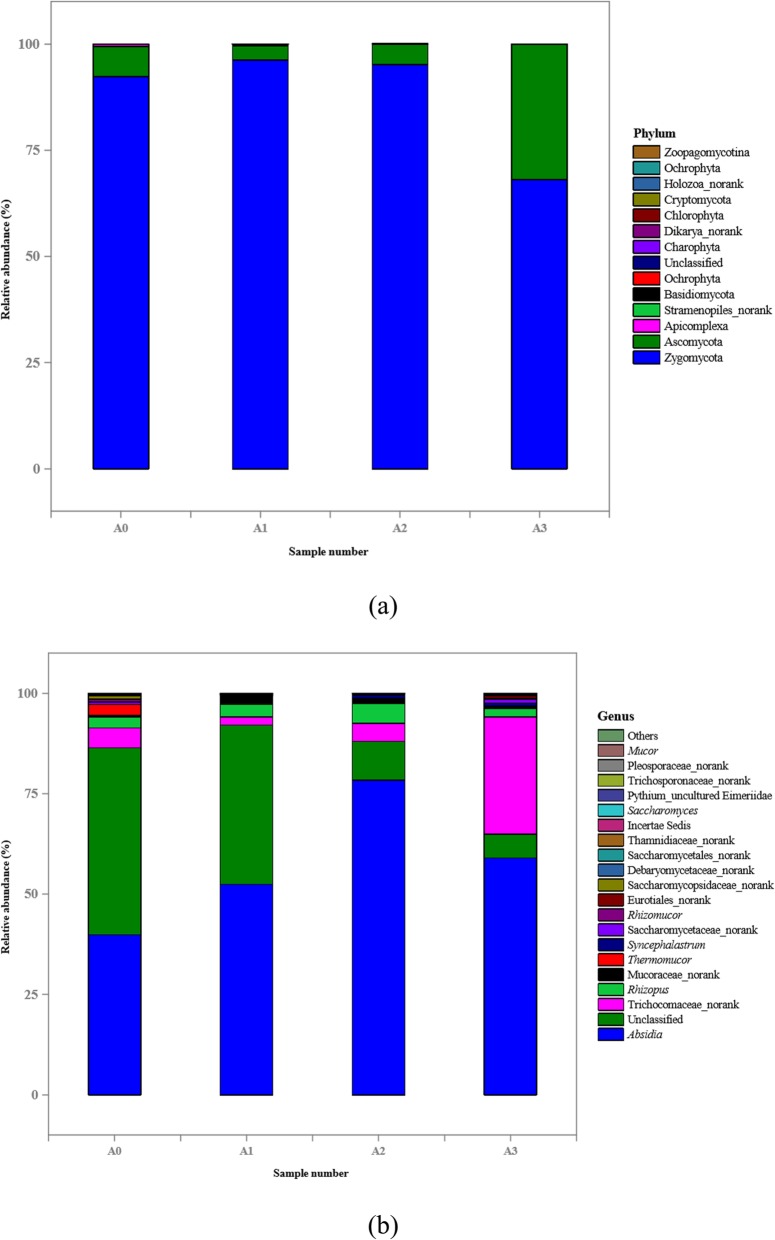
Analysis of eukaryotic communities composition in four samples (A0, at the beginning of aging; A1, after 1 month of aging; A2, after 2 months of aging; A3, after 3 months of aging) using high-throughput sequencing. Results of taxonomy at the phylum level and genus level were shown in (**a**) and (**b**), respectively. The relative abundance defines sequence percentages in samples as depicted by colors in the bar chart. The top 20 phyla and genera detected in RSF *Daqu* are shown in (**a**) and (**b**), respectively. The rest of phyla and genera detected in all samples were classified as others

At the phylum level, the predominant eukaryotic communities were Zygomycota and Ascomycota during the *Daqu* aging, which was consistent with previous reports [26]. In general, these two phyla constituted more than 99.0% of the total abundance during the entire aging process (Fig. 4a). However, their changes during aging showed opposite trends. In detail, Zygomycota was increased during the first two months of aging, and then decreased in the third month, while the pattern for Ascomycota was the opposite. These findings were similar to those in a previous report, where the abundance of Ascomycota and Zygomycota gradually approached each other at the end of aging, with some differences [14]. The same study showed that the population of Ascomycota was slightly higher than that of Zygomycota, perhaps due to the different manufacturing environments for these two *Daqus*, leading to a *Baijiu* that was different in style [14]. The eukaryotic community succession was investigated according to OTUs classified at the genus level (Fig. 4b). The results showed that *Absidia* and Unclassified were the dominant genera in all *Daqu* samples, with Trichocomaceae_norank and *Rhizopus* forming the subdominant genera. As aging proceeded, *Absidia* and *Rhizopus* initially increased for up to 2 months, and then declined at the end of aging, while Trichocomaceae_norank showed the opposite trend, becoming the predominant genus in mature *Daqu*. Interestingly, *Thermomucor*, which has only been reported in *Daqu* by Li and Qiu, was a subdominant genus at the beginning of aging, but had disappeared after 1 month [29]. This might have been due to the interaction between microbes in *Daqu* and the environment during aging. During aging, *Syncephalastrum* might be present in the environment because it was not present in *Daqu* at the beginning of aging and became almost a subdominant genus after aging. Therefore, environmental microbiota influenced the microbial succession during aging, meaning that they also played an important role in the maturation of *Daqu* during aging [25]. After aging, some rare genera, such as *Rhizomucor* and *Saccharomyces*, were observed.

Molds and yeasts are mainly responsible for saccharification and alcoholic fermentation in the production of *Baijiu*. In the present study, four mold and one yeast genera were identified in *Daqu*, namely, *Absidia*, *Rhizopus*, *Syncephalastrum*, *Rhizomucor*, and *Saccharomyces*. *Absidia* and *Rhizopus* were the most frequent and important fungi found in *Daqu* [6]. *Absidia*, a main amylase producer, has a strong saccharification capability and contributes significantly to *Baijiu* fermentation [30]. *Rhizopus* also secretes large amounts of various hydrolyzing enzymes that decompose macromolecules and produce smaller metabolites and volatile compounds, such as ethanol, 2-methyl-1-butanol, and 3-methyl-1-butanol, which affect the flavor of *Baijiu* [6, 30, 31]. *Syncephalastrum*, first detected in *Daqu*, has been shown to secrete enzymes, such as amylase, glucosidase and pullulanase, that degrade starch [32]. *Rhizomucor* is also considered to be a functional microbe, with a high ability to produce saccharifying amylase [3, 9]. Therefore, *Absidia*, *Rhizopus*, *Syncephalastrum*, and *Rhizomucor* all contribute to saccharifying and liquefying activities in *Daqu* although their activity decreased as aging progressed [3, 6]. This might be due to several factors, such as: The activity of enzymes that degrade starch into sugar is different in these genera; the fluctuation in the number of these microorganisms is inconsistent during the whole aging process; and, in addition to these genera, there are other microorganisms, such as bacteria and yeasts, which exhibit saccharifying and liquefying activities. *Saccharomyces* is the most abundant yeast and the most efficient ethanol producer during the *Baijiu* fermentation stage, with the *Baijiu* produced being associated with high scores for harmony, and the highest values of floral aroma compared with other yeasts from sensory evaluation [33, 34]. Although the numbers of *Saccharomyces* decreased during aging, there were no significant changes in fermentation capacity in the *Daqu* samples. This might have been due to the balance between the abundance of *Saccharomyces* and other yeasts, belonging to Saccharomycetaceae, Saccharomycopsidaceae, or Saccharomycetales, which have a certain ability to produce ethanol.

### Relationships between the physicochemical properties and microbial communities of *Daqu*

**Fig. 5:**
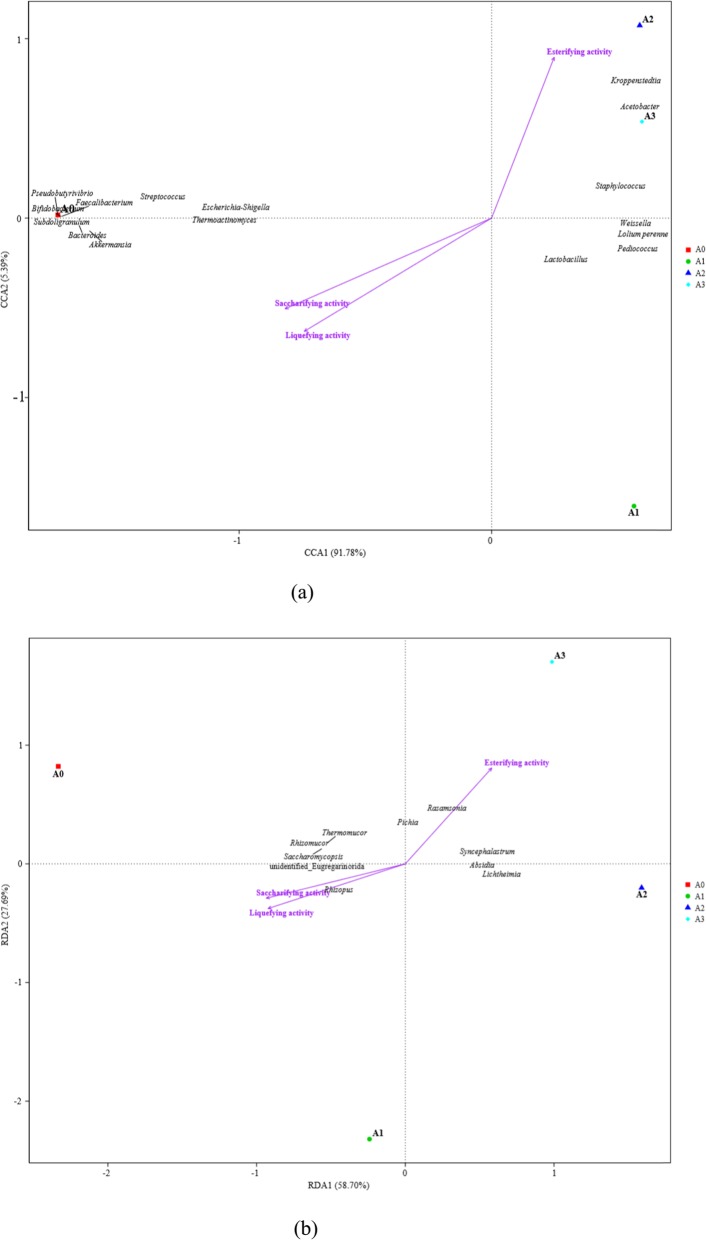
Canonical correspondence analysis (CCA) of prokaryotic communities at genus level and physicochemical properties (**a**), and redundancy discriminate analysis (RDA) of eukaryotic communities at genus level and physicochemical properties (**b**) found in *Daqu*. Arrows represent different physicochemical properties; the symbols ■, ●, ▲, and ◆ represent samples A0 (at the beginning of aging), A1 (after 1 month of aging), A2 (after 2 months of aging), and A3 (after 3 months of aging), respectively. The tow axes explain 97.17 and 86.39% of the total variance in bacterial (DCA = 4.10) and fungal (DCA = 1.15) community differentiation, respectively

Microbial communities generally have significant effects on some physicochemical properties. In the present study, RDA and CCA were performed based on the prokaryotic and eukaryotic communities, and the physicochemical properties (Fig. 5). Overall, the two axes explained 97.17 and 86.39% of the total variance in bacterial (Fig. 5a, DCA = 4.10) and fungal (Fig. 5b, DCA = 1.15) community differentiation, respectively, suggesting remarkable correlations between the microbial communities and the physicochemical properties. Figure 5a shows that *Pseudobutyrivibrio*, *Bifidobacterium*, *Subdoligranulum*, *Faecalibacterium*, *Bacteroides*, *Akkermansia*, *Streptococcus*, *Escherichia*-*Shigella*, and *Thermoactinomyces* were positively correlated with sample A0, which was consistent with previous results (Fig. 3). These genera were positively correlated with the saccharifying and liquefying activities (Fig. 5a). In previous reports, most of these microorganisms can produce glucosidase and amylase [35–43]. Remarkable positive correlations were also determined between *Kroppenstedtia*, *Acetobacter*, and *Staphylococcus* and samples A2 and A3. These genera contributed to the high esterifying activity of both samples, possibly because they can yield lipase or esterase (Fig. 5a) [44, 45]. Another possible explanation was that these bacteria can produce organic acids, which are transformed into esters by lipase or esterase produced by other microbes or esterification with ethanol [24, 28]. The RDA results showed a significant positive correlation between *Thermomucor*, *Rhizomucor*, *Saccharomycopsis*, Unidentified_Eugregarinorida, and *Rhizopus* and the saccharifying and liquefying activities, which further confirmed that the above strains were the main producers of amylase or glucosidase [3, 6, 31]. RDA also revealed that *Rasamsonia*, *Pichia*, *Syncephalastrum*, *Absidia*, and *Lichtheimia* were positively correlated with esterifying activity. Some studies have found that these genera can produce lipase or esterase, causing the high esterifying activity [46–49].

### Correlations between microbial communities and flavor compounds

**Fig. 6:**
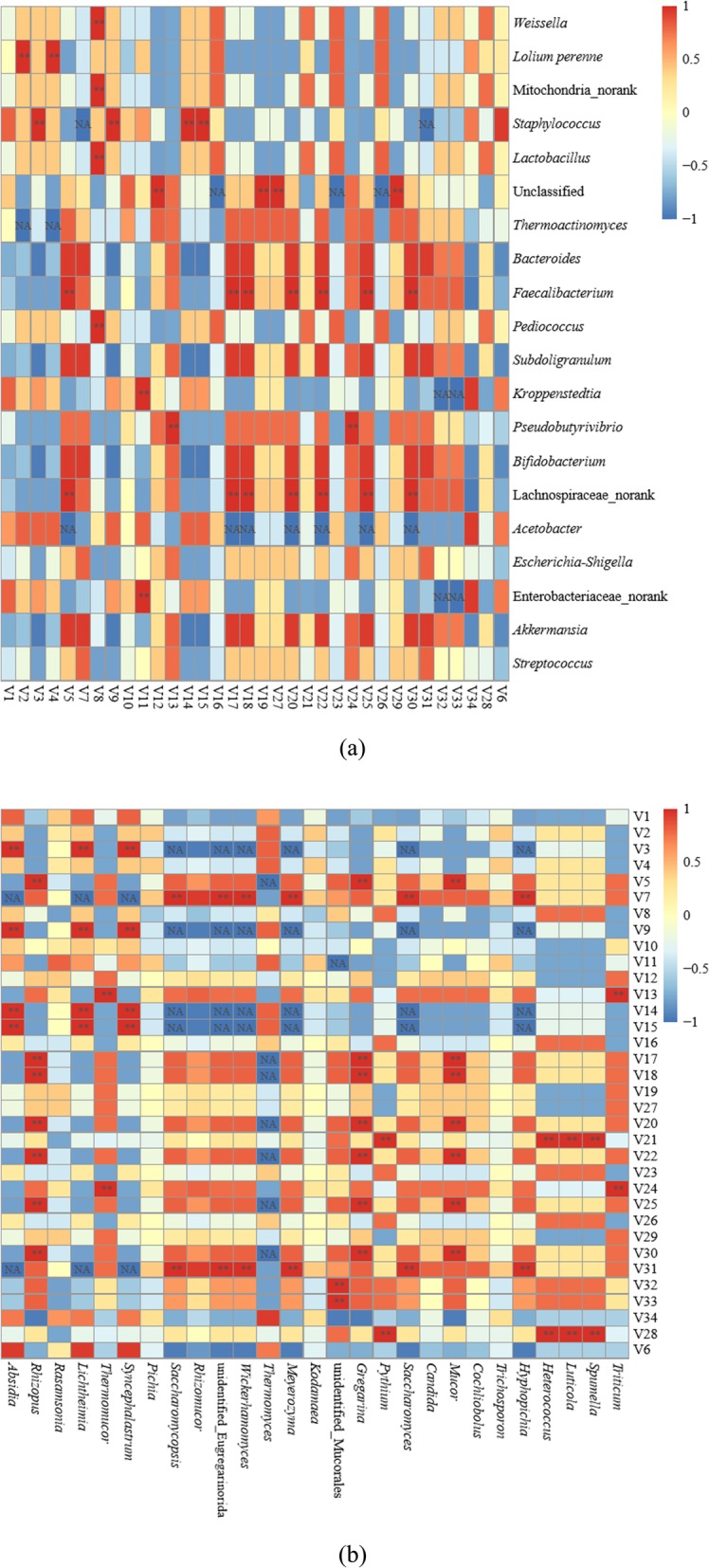
Correlation between volatile compounds (V1-V34: different flavor compounds in Additional file 1 Table S1) and prokaryotic communities (**a)** and eukaryotic communities (**b**) found in *Daqu*. Scale bar colors denote the nature of the correlation, with 1 indicating a perfectly positive correlation (read) and − 1 indicating a perfectly negative correlation (blue) between them. “*” shows significant correlations (*P* < 0.05), and “NA” shows no correlations

The heatmap revealed the correlations between the microbial communities and flavor compounds (Fig. 6). Ledol, a lipophilic aromatic compound, was positively correlated with *Staphylococcus*, *Absidia*, *Lichtheimia*, and *Syncephalastrum*. However, there have been no reports of these genera synthesizing or metabolizing the corresponding flavor substances. No report for *p*-toluic acid 2-ethylhexyl ester, and the analysis showed that this compound was positively correlated with eukaryotes, such as *Saccharomycopsis*, unidentified_Eugregarinorida, *Wickerhamomyces*, *Meyerozyma*, *Saccharomyces*, and *Hyphopichia*. In contrast, phthalic acid hept-4-yl isobutyl ester, a main compound in the flowers of *musk melon* (*Cucumis melo* L.) and roasted peanuts, was positively correlated with prokaryotes, such as *Weissella*, Mitochondria_norank, *Lactobacillus*, and *Pediococcus* [50, 51]. Higher fatty acid esters, such as 9,12-octadecadienoic acid ethyl ester, are colloidal substances that play a vital role in the stability and taste of *Baijiu* [52]. Figure 6 shows that Enterobacteriaceae_norank, and *Kroppenstedtia* were highly positively correlated with 9,12-octadecadienoic acid ethyl ester. However, there are no previous studies on this correlation and possible metabolism, which require further verification and study. Hexanal, associated with pleasant descriptors, such as herbal, fruity and flowery, was positively correlated with *Staphylococcus*, *Absidia*, *Lichtheimia*, and *Syncephalastrum*, which, except for *Lichtheimia*, were confirmed by previous results [53–55]. A previous report has shown that hexanal was oxidized from hexylamine by amine oxidases produced by the above genera [55]. Benzaldehyde was also positively correlated with *Staphylococcus*, *Absidia*, *Lichtheimia* and *Syncephalastrum*. However, except for the relationship between benzaldehyde and *Staphylococcus*, the relationships among others have not been reported [56]. Statistical analysis performed in the present study indicated a positive correlation between (E)-2-octenal/nonanal and *Faecalibacterium*, Lachnospiraceae_norank, *Rhizopus*, *Gregarina*, and *Mucor*. To our knowledge, only the relationship of (E)-2-octenal with *Rhizopus* and *Mucor* has been confirmed in previous studies, with the 2-alkenal reductase produced playing an important role in the synthesis of (E)-2-octenal [31, 57]. *Faecalibacterium*, Lachnospiraceae_norank, *Rhizopus*, *Gregarina*, and *Mucor* had an important influence on the compounds 2,2,4-trimethyl-1,3-pentanediol, 2-methyl-naphthalene, 1,7-dimethyl-naphthalene, 2-undecanone, and tetradecane. Their correlations and possible metabolism require further verification and study because no relevant studies have yet been reported. *Pythium*, *Heterococcus*, *Luticola*, and *Spumella* had positive loadings on 4-ethenyl-1,2-dimethoxy-benzene and (2-dodecen-1-yl) succinic anhydride from the heatmap. Alkanes pentadecane and hexadecane were positively correlated with unidentified_Mucorales, while 3-methyl-tetradecane was positively correlated with *Saccharomycopsis*, unidentified_Eugregarinorida, *Wickerhamomyces*, *Meyerozyma*, *Saccharomyces*, and *Hyphopichia* (Fig. 6). No studies have described these relationships previously.

## Conclusions

This study has comprehensively demonstrated the changes in the physicochemical properties, flavor components, and microbial communities of *Daqu* during aging. The physicochemical properties of *Daqu* changed significantly during aging. The higher liquefying and saccharifying activities of *Daqu* at the beginning of aging, changed to a high esterifying activity, and moderate liquefying and saccharifying activities, at the end of aging. The aging process improved both the ester content and fragrance of *Daqu*. These changes were undoubtedly caused by changes in the microbial communities in *Daqu*, which were attributed to the external environment and interaction with external microorganisms during aging. Overall, the results showed that physicochemical properties, such as acidity, and sensory factors, such as flavor components and appearance, can provide a scientific basis to determine whether *Daqu* is mature. However, the microbial community is essential for understanding the aging process when making *Daqu*. This study has provided a deeper theoretical basis to explain the need for aging when making *Daqu*, and the means to design effective strategies to manipulate the preparation of *Daqu* to better control *Baijiu* production systems and to improve the quality of the final product.

## Methods

### Sampling

Four samples of *Daqu* from the same production set were taken at different times until it was judged to be mature by experienced workers: A0, at the beginning of aging, sampled on May 8, 2017 (non-aging *Daqu*); A1, after 1 month of aging, sampled on June 8, 2017; A2, after 2 months of aging, sampled on July 8, 2017; A3, after 3 months of aging, sampled on August 8, 2017 (end aging *Daqu*). The samples were then collected from the liquor (RSF) at Shandong Bandaojing Co. Ltd. (Shandong, China). The bricks of *Daqu* were freshly produced from wheat grains during spring 2017. To obtain reliable samples for analysis, each sample was randomly selected from upper, middle, lower and inside locations in the storage room (Fig. 1), then promptly ground to a powder, mixed well to form one sample, and collected by quartering [58]. The samples were transferred to sterile bags, sealed, and then stored at − 80 °C for further analysis.

### Analysis of physicochemical properties

The physicochemical properties of the *Daqu* samples, namely, moisture, protein, and starch contents, acidity, pH, amino nitrogen (AN), reducing sugar (RS), and total ester (TE) levels, and saccharifying, liquefying, esterifying, and fermenting activities, were measured three times, as described in a previous report [14].

### Analysis of volatile compounds

The contents of volatile compounds in the *Daqu* samples were determined using HS-SPME-GC-MS (TSQ 8000 Evo, Trace MS/GC; Thermo Fisher Scientific, Waltham, MA, USA) as previously described by Fan et al. [59]. Individual volatile compounds were identified by comparison with data from the NIST 05a spectra library (Gaithersburg, MD, USA), then quantified according to the amount of internal standard (2-octanol, Sigma-Aldrich Chemical Co., USA) added to the *Daqu* sample before extraction and expressed as mg per kg of *Daqu*. The analyses were performed in triplicate.

### DNA extraction and quantitation

*Daqu* samples were prepared using a method described in a previous study [10]. A commercial PowerSoil DNA Isolation Kit (Mo Bio Laboratories Inc., Carlsbad, CA, USA) was used to extract the genomic DNA. After being quantified using spectrophotometry (ratio of optical densities at 260 and 280 nm) and electrophoresis in 0.6% (w/v) agarose gels, the genomic DNA was adjusted to equal concentrations for use as a template for PCR amplification.

### PCR amplification and Illumina MiSeq sequencing

The V3-V4 regions on the 16S ribosomal RNA gene of the bacteria were amplified with forward primers 340F (5′-barcode-CCTACGGGNBGCASCAG-3′) and reverse primers 805R (5′-GACTACNVGGGTATCTAATCC-3′). The 18S ribosomal RNA gene of the fungi was amplified using forward primer 512F (5′-TATTCCAGCTCCAATAGCG-3′) and reverse primer 978R (5′-barcode-GACTACGATGGTATCTAATC-3′). The primer was modified by adding an error-correcting barcode, which was unique to each sample and served as a multiplexing marker. The PCR reactions and extraction of amplicons were performed as described in a previous report [59]. After quantification using the QuantiFluor-ST system (Promega Corp., Fitchburg, WI, USA), the purified amplicons were pooled in equimolar amounts then paired-end sequenced (2 × 250 bp) on an Illumina HiSeq2500 sequencing platform (Illumina Inc., San Diego, CA, USA) in accordance with the standard protocols. The raw reads have been deposited in the NCBI Sequence Read Archive (SRA) database (SRP161533).

### Processing of sequencing data

The raw Illumina *fastq* files were demultiplexed, then quality-filtered using the QIIME (version 1.8) microbiome analysis software (quiime.org). Reads which could not be assembled were discarded, and chimeric sequences were identified and removed using UCHIME. The operational taxonomic units (OTUs) with ≥97% similarity were clustered using UPARSE (version 7.1, drive5.com/uparse/). The taxonomy of each 16S rRNA/18S rRNA gene sequence was obtained using RDP Classifier (rdp.cme.msu.edu/) against the Silva (SSU115) 16S rRNA and 18S rRNA databases with a confidence threshold of 70% (www.arb-silva.de).

The α-diversity, used to estimate the richness and diversity of OTUs, was calculated using QIIME to generate rarefaction curves, Good’s coverage, Chao 1, ACE, and Shannon and Simpson diversity indices. The β-diversity was evaluated using the UniFrac method. The weighted UniFrac distance matrices and OTU tables were calculated using QIIME to evaluate differences between the matrices and producers. The *group_significance.py* script of QIIME was run to compare the frequencies of OTUs across samples. Both the weighted and unweighted calculations were performed using principal coordinate analysis (PCoA). Redundancy analysis (RDA), canonical correspondence analysis (CCA), and heatmaps were performed using the *vegan* and *pheatmap* packages in R (vegan.r-forge.r-project.org/), to confirm the correlations between community structures and environmental variables, respectively.

### Statistical analysis

Each treatment was replicated three times. All statistical analyses were performed using SPSS_16.0 (SPSS Inc. Chicago, IL. USA). The significance of differences between mean values was examined using Tukey’s test. Differences between means at *P* < 0.05 were considered significant.

## Supplementary information

**Additional file 1: Table S1.** Volatile compounds identified in RSF *Daqu* samples.

**Additional file 2: Figure S1.** The clustering analysis was performed using UPGMA, which is a type of hierarchical clustering method based on unweighted UniFrac distance metrics, and showed the relationship of the prokaryotic communities in the four samples. **Figure S2.** PCoA analysis of the prokaryotic communities in the four samples. **Figure S3.** The clustering analysis was performed using UPGMA, which is a type of hierarchical clustering method based on unweighted UniFrac distance metrics, and showed the relationship of the eukaryotic communities in the four samples. **Figure S4.** PCoA analysis of the eukaryotic communities in the four samples.

## Data Availability

The data generated or analyzed during this study are included in this published article and its Additional file. The original sequencing data supporting the conclusions of this article is available in the National Center for Biotechnology Information Sequence Read Archive (SRA) database, accession no. SRP161533 and hyperlink to dataset in https://www.ncbi.nlm.nih.gov/sra/SRP161533.

## Abbreviations

AN: Amino nitrogen
CCA: Canonical correspondence analysis
HS-SPME-GC-MS: Headspace solid-phase microextraction-gas chromatography-mass spectrometry
HTS: High-throughput sequencing
LAB: Lactic acid bacteria
OTUs: Operational taxonomic units
PCoA: Principal coordinate analysis
RDA: Redundancy analysis
RS: Reducing sugar
RSF: Roasted sesame-like flavor
TE: Total ester
