# Supplementary material for: Effects of aging on the quality of roasted sesame-like flavor Daqu
Source: BMC Microbiol. 2020 Mar 26;20:67. doi: 10.1186/s12866-020-01745-3 (PMC7098102; doi:10.1186/s12866-020-01745-3)
Supplement: Supplementary file 1 — Additional file 1: Table S1. Volatile compounds identified in RSF Daqu samples. [file 12866_2020_1745_MOESM1_ESM.pdf]

Table

Table S1 Volatile compounds identified in RSF *Daqu* samples.

Code	Compounds	Concentration (mg/kg)			
		A0	A1	A2	A3
***Alcohols* (6)**					
V1	1-Octen-3-ol	0.0215±0.0028^{b}	0.0110±0.0027^{a}	0.0398±0.0010^{c}	0.0386±0.0007^{c}
V2	Phenylethyl alcohol	0.2298±0.0309^{a}	0.3928±0.0719^{b}	0.3783±0.0390^{b}	0.4081±0.0031^{b}
V3	Ledol	0.1085±0.0150^{a}	0.1135±0.0274^{ab}	0.1368±0.0008^{b}	0.1327±0.0050^{b}
V4	1-Pentanol, 4-methyl-	0.0147±0.0012^{a}	0.0188±0.0011^{bc}	0.0184±0.0001^{b}	0.0204±0.0028^{bc}
V5	2,2,4-Trimethyl-1,3-pentanediol	0.0260±0.0031^{c}	0.0195±0.0027^{b}	0.0148±0.0005^{a}	0.0136±0.0012^{a}
V6	2,4,7,9-Tetramethyl-5-decyn-4,7-diol	nd*	nd	0.0161±0.0001^{a}	0.0156±0.0005^{a}
Σ		**0.4005**	**0.5556**	**0.6042**	**0.6290**
***Esters* (7)**					
V7	p-Toluic acid, 2-ethylhexyl ester	0.0095±0.0003^{b}	0.0080±0.0015^{b}	0.0054±0.0010^{a}	0.0061±0.0001^{a}
V8	Phthalic acid, hept-4-yl isobutyl ester	0.0661±0.0086^{a}	0.0741±0.0120^{a}	0.0740±0.0011^{a}	0.0728±0.0006^{a}
V9	Dibutyl phthalate	0.1101±0.0203^{a}	0.1189±0.0063^{a}	0.1375±0.0113^{a}	0.1228±0.0096^{a}
V10	Hexadecanoic acid, ethyl ester	0.0631±0.0183^{ab}	0.0562±0.0061^{a}	0.0668±0.0008^{b}	0.0608±0.0077^{a}
V11	9,12-Octadecadienoic acid, ethyl ester	0.0387±0.0130^{ab}	0.0313±0.0027^{a}	0.0390±0.0010^{b}	0.0413±0.0042^{b}
V12	(E)-9-Octadecenoic acid ethyl ester	0.0456±0.0178^{ab}	0.0266±0.0072^{a}	0.0418±0.0076^{b}	0.0359±0.0008^{b}
V13	2-Propanol, 1-chloro-, phosphate (3:1)	0.0082±0.0020	nd	nd	nd
Σ		**0.3413**	**0.3151**	**0.3645**	**0.3397**
***Aldehydes* (6)**					
V14	Hexanal	0.0378±0.0079^{a}	0.1157±0.0194^{b}	0.1358±0.0156^{b}	0.1218±0.0043^{b}
V15	Benzaldehyde	0.0406±0.0032^{a}	0.0530±0.0194^{ab}	0.0595±0.0011^{b}	0.0590±0.0005^{b}
V16	Benzeneacetaldehyde	0.0628±0.0067^{a}	0.0700±0.0026^{a}	0.0657±0.0022^{a}	0.0673±0.0010^{a}
V17	2-Octenal, (E)-	0.0249±0.0063^{b}	0.0208±0.0002^{b}	0.0159±0.0006^{a}	0.0151±0.0005^{a}
V18	Nonanal	0.0287±0.0054^{b}	0.0276±0.0036^{b}	0.0158±0.0003^{a}	0.0149±0.0009^{a}
V19	2-Nonenal, (E)-	0.0402±0.0214^{b}	0.0126±0.0032^{a}	0.0170±0.0014^{a}	0.0152±0.0010^{a}

**Σ**		**0.2350**	**0.2997**	**0.3097**	**0.2933**
***Benzodiazepines* (3)**					
V20	Naphthalene, 2-methyl-	0.0217±0.0003^{b}	0.0153±0.0028^{a}	0.0152±0.0003^{a}	0.0144±0.0009^{a}
V21	Benzene, 4-ethenyl-1,2-dimethoxy-	nd	0.012±0.0031	nd	nd
V22	Naphthalene, 1,7-dimethyl-	0.0302±0.0025^{b}	0.0242±0.0096^{b}	0.0078±0.0001^{a}	0.0076±0.0003^{a}
**Σ**		**0.0519**	**0.0515**	**0.0230**	**0.0220**
***Nitrogen-containing compounds* (1)**					
V23	Pyrazine, tetramethyl-	0.0199±0.0027^{a}	0.0790±0.0195^{d}	0.0339±0.0096^{b}	0.0577±0.0059^{cd}
Ketones (5)					
V24	Acetophenone	0.0084±0.0022	nd	nd	nd
V25	2-Undecanone	0.0170±0.0038^{b}	0.0128±0.0033^{ab}	0.0101±0.0008^{a}	0.0088±0.0011^{a}
V26	2-Pentadecanone, 6,10,14-trimethyl-	nd	0.0072±0.0005^{a}	0.0069±0.0002^{a}	0.0071±0.0005^{a}
V27	2(3H)-Furanone, dihydro-5-pentyl-	0.0136±0.0004^{b}	nd	0.0089±0.0005^{a}	0.0085±0.0001^{a}
V28	2-Dodecen-1-yl(-)succinic anhydride	nd	0.0092±0.0044	nd	nd
**Σ**		**0.0390**	**0.0292**	**0.0259**	**0.0244**
***Phenols* (1)**					
V29	2-Methoxy-4-vinylphenol	0.0381±0.0084^{b}	nd	0.0199±0.0034^{a}	0.0181±0.0008^{a}
***Alkane* (4)**					
V30	Tetradecane	0.0269±0.0025^{c}	0.0217±0.0029^{b}	0.0113±0.0006^{a}	0.0108±0.0002^{a}
V31	Tetradecane, 3-methyl-	0.0219±0.0016^{b}	0.0208±0.0032^{b}	0.0044±0.0002^{a}	0.0045±0.0001^{a}
V32	Pentadecane	0.0265±0.0024^{b}	0.0266±0.0053^{b}	0.0115±0.0004^{a}	0.0109±0.0004^{a}
V33	Hexadecane	0.0113±0.0010^{bc}	0.0132±0.0012^{c}	0.0099±0.0013^{ab}	0.0084±0.0008^{a}
**Σ**		**0.0866**	**0.0823**	**0.0371**	**0.0346**
***Alkene* (1)**					
V34	Caryophyllene	nd	nd	0.0207±0.0006^{a}	0.0236±0.0047^{b}

Note: Same letters in the column do not differ significantly at 5% probability by Tukey test. A0, at the beginning of aging; A1, after one month of aging; A2, after two months of aging; A3, after three months of aging. * nd, not detected.
