# Supplementary material for: Effects of aging on the quality of roasted sesame-like flavor Daqu
Source: BMC Microbiol. 2020 Mar 26;20:67. doi: 10.1186/s12866-020-01745-3 (PMC7098102; doi:10.1186/s12866-020-01745-3)
Supplement: Supplementary file 2 — Additional file 2: Figure S1. The clustering analysis was performed using UPGMA, which is a type of hierarchical clustering method based on unweighted UniFrac distance metrics, and showed the relationship of the prokaryotic communities in the four samples. Figure S2. PCoA analysis of the prokaryotic communities in the four samples. Figure S3. The clustering analysis was performed using UPGMA, which is a type of hierarchical clustering method based on unweighted UniFrac distance metrics, and showed the relationship of the eukaryotic communities in the four samples. Figure S4. PCoA analysis of the eukaryotic communities in the four samples. [file 12866_2020_1745_MOESM2_ESM.pdf]

Figure legends

Fig. S1. The clustering analysis was performed using UPGMA, which is a type of hierarchical clustering method based on unweighted UniFrac distance metrics, and showed the relationship of the prokaryotic communities in the four samples (A0, at the beginning of aging; A1, after one month of aging; A2, after two months of aging; A3, after three months of aging).

Fig. S2. PCoA analysis of the prokaryotic communities in the four samples (A0, at the beginning of aging; A1, after one month of aging; A2, after two months of aging; A3, after three months of aging). The first two principal components (PC1 and PC2) can explain 80.96% of the data variance.

Fig. S3. The clustering analysis was performed using UPGMA, which is a type of hierarchical clustering method based on unweighted UniFrac distance metrics, and showed the relationship of the eukaryotic communities in the four samples (A0, at the beginning of aging; A1, after one month of aging; A2, after two months of aging; A3, after three months of aging).

Fig. S4. PCoA analysis of the eukaryotic communities in the four samples (A0, at the beginning of aging; A1, after one month of aging; A2, after two months of aging; A3, after three months of aging). The first two principal components (PC1 and PC2) can explain 74.88% of the data variance

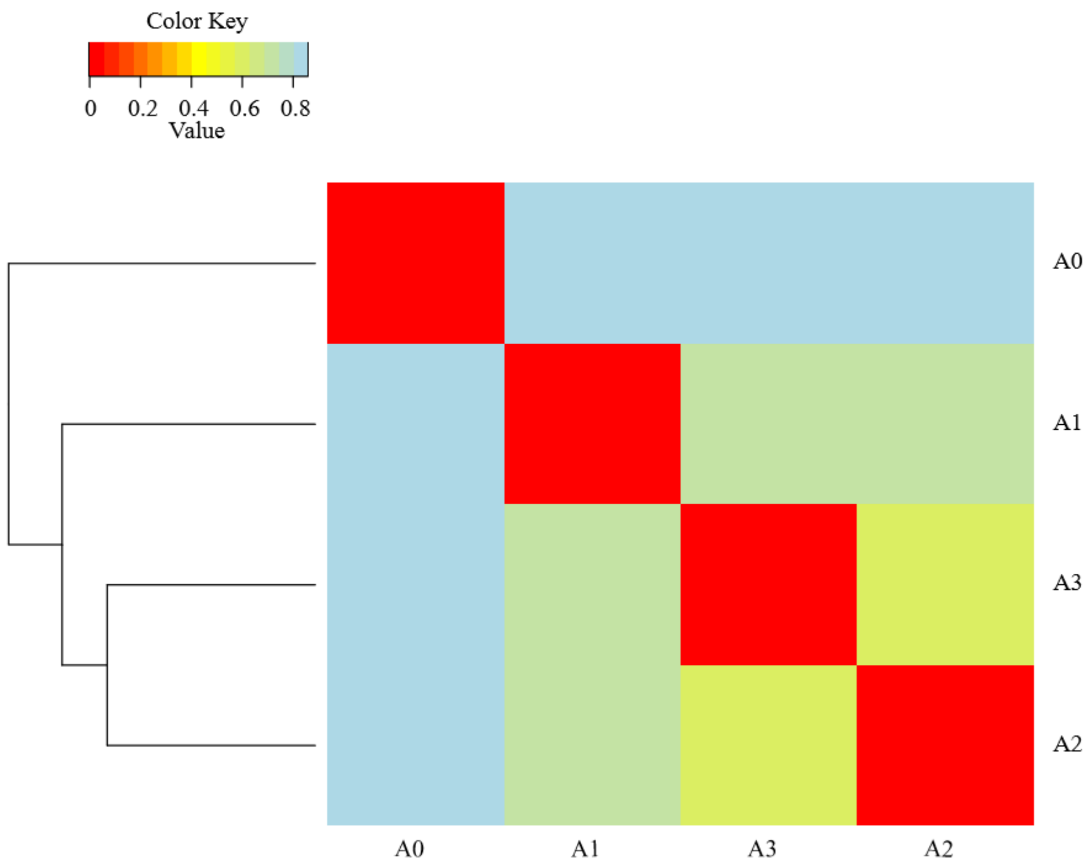

Fig. S1

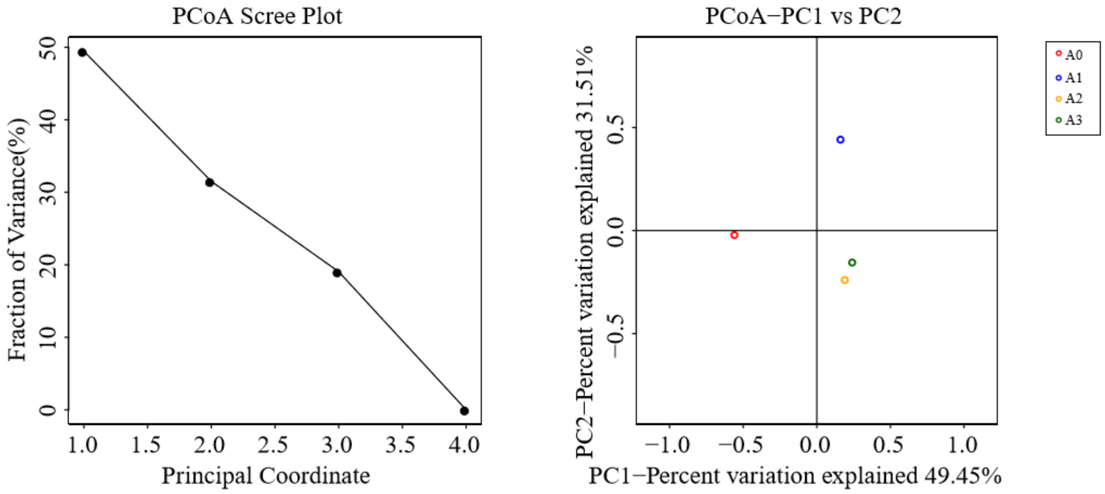
PCoA Scree Plot
Fraction of Variance(%)
Principal Coordinate
PCoA−PC1 vs PC2
PC2−Percent variation explained 31.51%
PC1−Percent variation explained 49.45%
A0
A1
A2
A3

Fig. S2

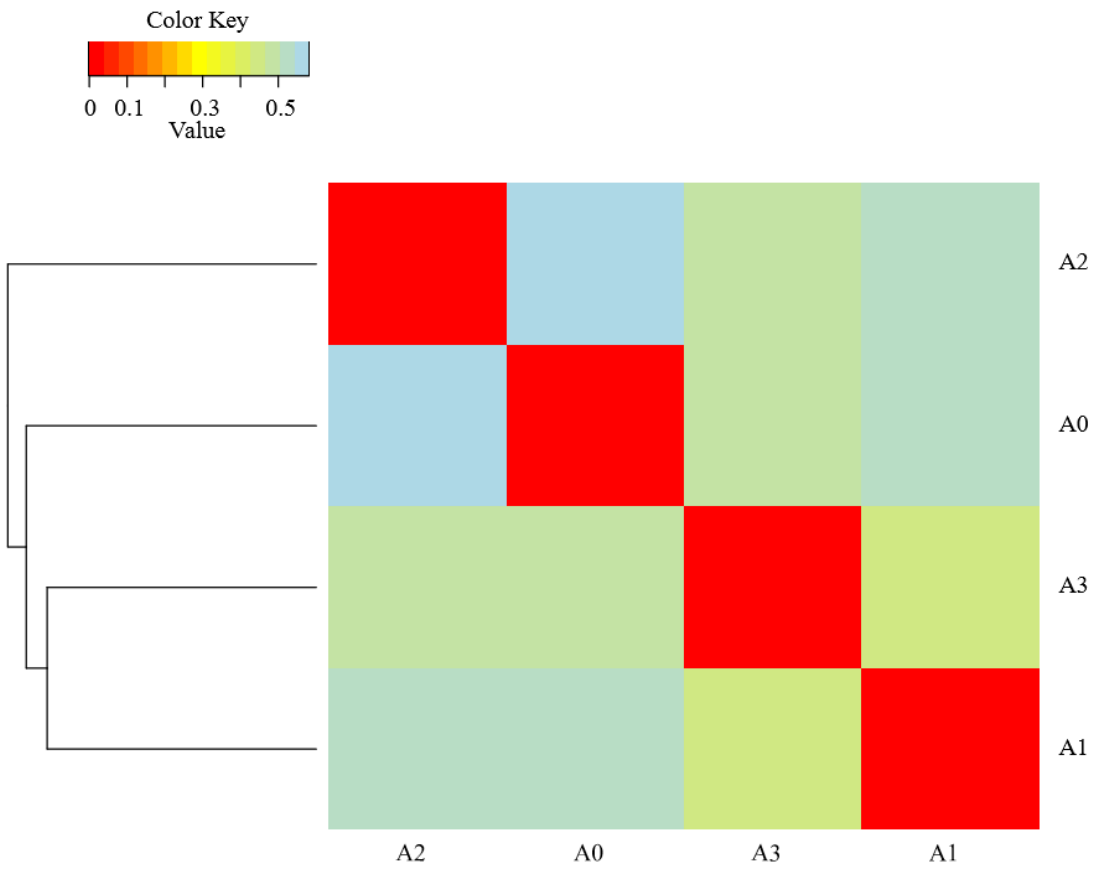

Fig. S3

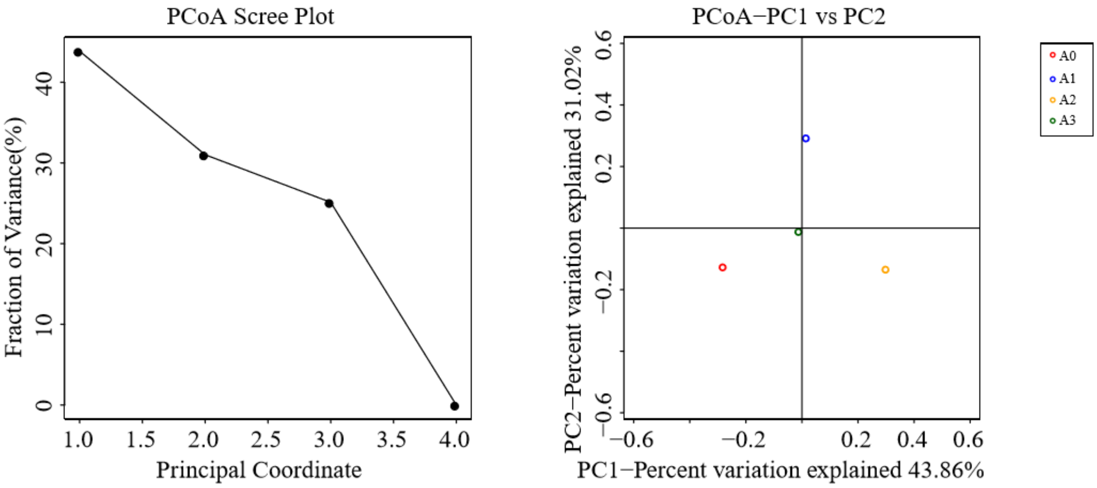
PCoA Scree Plot
Fraction of Variance(%)
Principal Coordinate
PCoA−PC1 vs PC2
PC2−Percent variation explained 31.02%
PC1−Percent variation explained 43.86%
A0
A1
A2
A3

Fig. S4
